# Expanded renal lymphatics improve recovery following kidney injury

**DOI:** 10.14814/phy2.15094

**Published:** 2021-11-21

**Authors:** Gaurav Baranwal, Heidi A. Creed, Laurence M. Black, Alexa Auger, Alexander M. Quach, Rahul Vegiraju, Han E. Eckenrode, Anupam Agarwal, Joseph M. Rutkowski

**Affiliations:** ^1^ Division of Lymphatic Biology Department of Medical Physiology Texas A&M University College of Medicine Bryan Texas USA; ^2^ Department of Medicine University of Alabama at Birmingham Birmingham Alabama USA; ^3^ Nephrology Research and Training Center University of Alabama at Birmingham Birmingham Alabama USA; ^4^ Department of Veterans Affairs Birmingham Veterans Administration Medical Center Birmingham Alabama USA

**Keywords:** AKI, lymphangiogenesis, POD‐ATTAC, Proteinuria, VEGF‐D, VEGFR‐3

## Abstract

Acute kidney injury (AKI) is a major cause of patient mortality and a major risk multiplier for the progression to chronic kidney disease (CKD). The mechanism of the AKI to CKD transition is complex but is likely mediated by the extent and length of the inflammatory response following the initial injury. Lymphatic vessels help to maintain tissue homeostasis through fluid, macromolecule, and immune modulation. Increased lymphatic growth, or lymphangiogenesis, often occurs during inflammation and plays a role in acute and chronic disease processes. What roles renal lymphatics and lymphangiogenesis play in AKI recovery and CKD progression remains largely unknown. To determine if the increased lymphatic density is protective in the response to kidney injury, we utilized a transgenic mouse model with inducible, kidney‐specific overexpression of the lymphangiogenic protein vascular endothelial growth factor‐D to expand renal lymphatics. “KidVD” mouse kidneys were injured using inducible podocyte apoptosis and proteinuria (POD‐ATTAC) or bilateral ischemia reperfusion. In the acute injury phase of both models, KidVD mice demonstrated a similar loss of function measured by serum creatinine and glomerular filtration rate compared to their littermates. While the initial inflammatory response was similar, KidVD mice demonstrated a shift toward more CD4+ and fewer CD8+ T cells in the kidney. Reduced collagen deposition and improved functional recovery over time was also identified in KidVD mice. In KidVD‐POD‐ATTAC mice, an increased number of podocytes were counted at 28 days post‐injury. These data demonstrate that increased lymphatic density prior to injury alters the injury recovery response and affords protection from CKD progression.

## Graphical abstract

Increased renal lymphatic density prior to kidney injury alters the inflammatory response. This appears to improve injury resolution beyond the acute phase, reducing fibrosis and the AKI to CKD progression.

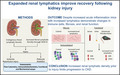

## 1 INTRODUCTION

Acute kidney injury (AKI) is a serious health condition associated with increased patient mortality, particularly in hospitalized patients. AKI is characterized by a sudden decline in renal function that is indicated by an increase in serum creatinine, reduction in glomerular filtration rate (GFR), or decrease in urine output over a 24–48 h period. AKI can rapidly progress to renal failure (Brown et al., 2016; Koyner et al., 2014; Pavkov et al., 2018). While many patients demonstrate short‐term recovery of renal function, clinical data suggest that AKI patients remain at an increased risk for the development of chronic kidney disease (CKD) and end‐stage renal disease due to maladaptive repair processes and sustained renal inflammation (Gameiro et al., 2021; Hsu et al., 2009; Humphreys et al., 2010; Sato & Yanagita, 2018). Several factors such as the nature of injury, rate of renal functional decline, microvascular rarefaction, mitochondrial dysfunction, immune infiltration, and interstitial fibrosis have been identified as having roles in the AKI‐to‐CKD transition (Basile et al., 2016; Black et al., 2019; Chou et al., 2018; Eardley et al., 2008; Fiorentino et al., 2018; Humphreys et al., 2010; Jiang et al., 2020; Sato & Yanagita, 2018; Tan et al., 2016; Yang et al., 2011; Zarjou et al., 2019). Another factor that may impact the AKI‐to‐CKD transition is the renal lymphatic vasculature.

In a healthy kidney, the renal lymphatic vasculature consists of a subcapsular network and lymphatic vessels that track along the larger interlobular and corticomedullary blood vessels (Breslin et al., 2018; Zarjou et al., 2019). Lymphatic vessel hyperplasia, expression of lymphatic growth factors, and de novo lymphangiogenesis have been reported in multiple forms of clinical and pre‐clinical AKI (Black et al., 2019; Karlsen et al., 2018; Zarjou et al., 2019). Whether lymphatic vessels and lymphangiogenesis play positive or negative roles following kidney injury is still unclear (Creed & Rutkowski, 2021). Historically, lymphatic vessels and lymphatic endothelial cells (LECs) were thought to be a purely passive system; however, recent research has increasingly demonstrated that lymphatics have active immunomodulatory roles that may impact inflammation resolution (Petrova & Koh, 2020).

Lymphatic endothelial cells, in cancer and other injury models, have been shown to regulate T cell activity and numbers through direct interaction with CD4+ and CD8+ T cells (Hirosue et al., 2014; Petrova & Koh, 2020; Vokali et al., 2020). In the context of AKI, research has demonstrated that various T cell populations may both mitigate and propagate the renal inflammatory response. For example, in cisplatin‐induced AKI, CD4+CD25+ T regulatory cells are reported to have protective roles while CD8+ T cells may increase injury (Lee et al., 2010; Liu et al., 2006). With the critical physiological roles of lymphatic vessels in regulating inflammation in other tissues and their recently defined active immunomodulatory roles, it is likely that lymphatic vessels play a role in the post‐AKI inflammatory response and chronic disease progression.

Our group has demonstrated that inducible kidney overexpression of the lymphangiogenic protein vascular endothelial factor‐D (VEGF‐D) results in renal lymphatic expansion, reduced intrarenal immune cell numbers, altered sodium excretion, and decreased blood pressure in hypertension (Balasubbramanian, Baranwal, et al., 2020; Balasubbramanian, Gelston, et al., 2020; Lopez Gelston et al., 2018). Accordingly, in the present study, we hypothesized that an increased renal lymphatic density would be protective following kidney injury. To test this hypothesis, we utilized a transgenic mouse model of kidney‐specific overexpression of VEGF‐D (KidVD) (Balasubbramanian, Baranwal, et al., 2020; Balasubbramanian, Gelston, et al., 2020; Lammoglia et al., 2016; Lopez Gelston et al., 2018) and induced renal lymphatic expansion prior to injury in two models of injury to the kidney: the POD‐ATTAC model of induced proteinuria by selective podocyte ablation (Dizin et al., 2020; Rutkowski et al., 2013) or bilateral renal ischemia‐reperfusion injury (IRI). While the initial extent of injury and functional loss was comparable to wild‐type injured littermates during the acute phase, KidVD mice displayed an altered immune response and decreased fibrosis suggesting a protective role of lymphatic vessel expansion to potentially reduce CKD progression.

## 2 MATERIALS AND METHODS

### 2.1 Mouse models

Generation and use of the KidVD mouse line (KSP‐rtTA × TRE‐VEGFD) have been previously described (Lammoglia et al., 2016; Lopez Gelston et al., 2018). POD‐ATTAC mice have been previously described and employed in injury and nephrotic syndrome studies (Dizin et al., 2020; Rutkowski et al., 2013). KidVD and POD‐ATTAC hemizygous mice were maintained on a C57/Bl6J lineage and crossed to generate experimental lines. Littermates lacking the necessary transgenes served as controls for both dimerizer and doxycycline administration: KidVD+PODO−, KidVD−PODO+, and KidVD−PODO−. Male mice aged 8–12 weeks at the start of VEGF‐D induction were utilized. Mice were provided ad libitum access to standard chow and drinking water throughout the study. All animal protocols were approved by the Institutional Animal Care and Use Committee at Texas A&M University or the University of Alabama at Birmingham (UAB) Institutional Animal Care and Use Committee.

### 2.2 Doxycycline administration and podocyte injury

Littermates of 8–12 weeks of age received doxycycline hyclate (0.2 mg/ml; Sigma) for 3 weeks in drinking water, followed by 1 day of normal water prior to induction of kidney injury. To activate Caspase‐8 driven apoptosis in podocytes, the dimerizer agent AP20187 was prepared according to the manufacturer's recommendation (Takara Bio USA, Inc.) and injected intraperitoneally at a dose of 0.4 µg/g body weight. All mice received a dimerizer injection. Injury was confirmed in POD+mice with observation of ascites at 2–3 days post‐injury (Rutkowski et al., 2013).

### 2.3 Bilateral renal IRI

KidVD mice and their littermates were provided 0.2 mg/ml doxycycline hyclate water for three weeks followed by 2 days of normal drinking water. Bilateral renal IRI was performed at the UAB‐UCSD O’Brien Center for Acute Kidney Injury Research (DK079337) and by Texas A&M College of Veterinary Medicine & Biomedical Sciences staff veterinarians as previously described (Zarjou et al., 2019). Mice were anesthetized by intraperitoneal injection of ketamine/xylazine, after which both renal pedicles were exposed and clamped for 20 min with atraumatic vascular clamps (Fine Science Tools; 18055‐05). Ischemia was confirmed by a rapid loss of color to the kidneys. Mice were maintained at 37°C throughout the procedure. After the clamps were removed, reperfusion was confirmed by the restoration of color before suturing. Sham‐operated mice had both renal pedicles exposed without clamp application.

### 2.4 Urine collection and analysis

Mice were placed into individual diuresis metabolic chambers (Hatteras Instruments) and provided *ad libitum* powdered chow and water for overnight acclimatization. Urine was subsequently collected over a 24‐h period after injection and prior to study termination. Twenty‐four hours urine volumes were measured and urine was briefly spun at a low speed to remove debris. Total urine protein concentrations were determined using a BCA assay kit (Pierce TM BCA Protein Assay Kit, Prod# 23225; Thermo Scientific); with sample dilutions of 1:40–1:400 with phosphate‐buffered saline (PBS). Specific urine albumin concentrations were determined using an Albuwell M kit (Exocell), with urine diluted 1:20–1:500, as necessary, in assay buffer. Urine creatinine analyses were conducted by the UAB‐UCSD O’Brien Center for Acute Kidney Injury Research using liquid chromatography‐tandem mass spectrometry.

### 2.5 Serum analysis

Creatinine values were measured as in urine and used to calculate creatinine clearance. Blood urea nitrogen (BUN) was quantified using Infinity Urea Liquid Stable Reagent (Thermo Fisher Scientific; TR12421) according to the manufacturer's instruction.

### 2.6 Transcutaneous GFR measurement

Glomerular filtration rate was measured in mice using the MediBeacon Transdermal GFR Measurement System (Scarfe et al., 2018). As previously described, mice were briefly anesthetized with isoflurane (1.5%–2% induction, 1%–1.5% maintenance) to shave and place transdermal GFR monitors on the flank region using a double‐sided adhesive patch (MediBeacon) (Black et al., 2018). Devices were secured to the mice using medical tape. Mice were allowed to recover from isoflurane anesthesia before intravenous fluorescein isothiocyanate (FITC)‐sinistrin injection (0.07–0.15 mg/g body weight). GFR was measured over a 1.5–2 h period in conscious mice with access to food and water ad libitum. Devices were then removed, and data were analyzed using elimination kinetics of FITC‐sinistrin clearance in MediBeacon MB Lab2 software. Mice utilized for transcutaneous GFR were not used for flow cytometry experiments to avoid a potential FITC signal.

### 2.7 Flow cytometry

#### 2.7.1 POD‐ATTAC studies

Whole de‐capsulated right kidneys were digested using Multi Tissue Dissociation Kit 2 on a gentleMacs Octa Dissociator (Miltenyi Biotec). Single‐cell suspensions were obtained by filtering the digested tissue through sterile 100 and 40 μm strainers. Red blood cells were lysed using ACK lysis buffer (Invitrogen; A1049201). Cells were re‐suspended in a 0.1% bovine serum albumin (BSA) solution and nonspecific Fc binding was blocked with an anti‐mouse CD16/CD32 antibody (BD Pharmingen) for 10 min on ice. After the blocking step, cells were incubated with fluorescent‐conjugated antibodies against CD45, F4/80, CD11c, CD19, Ly6G, CD3e, CD8a, and CD4 for 20 min on ice (Table 1). Data were acquired on a BD LSR Fortessa X‐20 flow cytometer using FACS DIVA software (BD Biosciences). Absolute number of CD45+ cells were normalized the total tissue mass and represented as CD45+ population/gram of tissue, while other cells were represented as percentage of CD45+ cells.

**TABLE 1:** List of antibodies utilized in this study

Antibody	Fluorophore	Clone	Manufacturer	Item number	RRID
CD45.2	FITC	104	BD Pharmigen	553772	AB_395041
CD45.2	PerCP‐Cy5.5	104	BD Biosciences	552950	AB_394528
CD45.1	Brilliant Violet 650	A20	Biolegend	110736	AB_2562564
CD45.2	Brilliant Violet 650	104	Biolegend	109836	AB_2563065
CD3e	Pacific Blue	500A2	BD Biosciences	558214	AB_397063
CD3e	PE‐Cy7	145‐2C11	Thermo Fisher	25‐0031‐82	AB_469572
CD3e	FITC	145‐2C11	Thermo Fisher	11‐0031‐82	AB_464882
CD19	APC	1 D3	BD Biosciences	557655	AB_396770
CD19	PE‐Cy7	eBio1D3	Thermo Fisher	25‐0193‐82	AB_657663
CD19	Brilliant Violet 785	6D5	Biolegend	115543	AB_11218994
CD16/32	n/a	2.4G2	BD Biosciences	553141	AB_394656
CD16/32	n/a	FRC‐4G8	Thermo Fisher	MFCR00‐4	AB_2539705
CD4	PE/Cy5	RM4‐5	Biolegend	100514	AB_312717
CD4	Super Bright 600	RM4‐5	Thermo Fisher	63‐0042‐82	AB_2637461
CD8a	PE	5306.7	Biolegend	100708	AB_312747
CD8a	eFluor 450	53‐6.7	Thermo Fisher	48‐0081‐82	AB_1272198
F4/80	PE/Cy7	BM8	Biolegend	123114	AB_893478
F4/80	APC eFluor 780	BM8	Thermo Fisher	47‐4801‐82	AB_2735036
Ly6G	APC	1A8	Thermo Fisher Scientific	17966882	AB_2573307
Ly6G	APC eFluor 780	RB6‐8C5	Thermo Fisher	47‐5931‐80	AB_1518805
Ly6C	eFluor 450	HK1.4	Thermo Fisher	48‐5932‐82	AB_10805519
CD11c	PerCP‐Cy 5.5	N418	Thermo Fisher Scientific	45‐0114‐80	AB_925728
CD11c	Brilliant Violet 785	N418	Biolegend	117336	AB_2565268
CD11b	Super Bright 600	M1/70	Thermo Fisher	63‐0112‐80	AB_2637407
WT1	n/a	F‐6	Santa Cruz Biotechnology	sc‐7385	AB_628448
LYVE‐1	n/a	n/a	R&D Systems	AF2125	AB_2297188
Podoplanin	n/a	n/a	R&D Systems	AF3244	AB_2268062
MHCII	FITC	M5/114.15.2	Thermo Fisher	11‐5321‐82	AB_465232
MHCII	APC	M5/114.15.2	Thermo Fisher	17‐5321‐82	AB_469455
Gr‐1	APC	1A8‐Ly6G	Thermo Fisher	17‐9668‐82	AB_2573307
Gr‐1	PE‐Cy7	1A8‐Ly6g	Thermo Fisher	25‐9668‐82	AB_2811793

#### 2.7.2 IRI studies

Mice were anesthetized with isoflurane (2.5% v/v induction, 1.5% v/v maintenance), and perfused with 10 ml of ice‐cold saline. De‐capsulated kidneys were minced and digested in Liberase DL (Roche) in Dulbecco's Modified Eagle's Medium, 10 mM HEPES, pH 7.4 at 37°C for 30 min while shaking. Ice‐cold isolation buffer (1% v/v BSA, 2 mM ethylenediaminetetraaceticacid [EDTA] in 1× PBS) was added to stop the enzymatic reaction. Tissues were disaggregated using 18‐G and 20‐G needles, followed by passage through a 40 μm filter and centrifuged. Red blood cells were lysed using ACK lysis buffer. Kidneys were washed in staining buffer (0.5% BSA, 0.01% sodium azide, 1× PBS) followed by Fcγr2/3 block (Clone 93) and subsequent staining for myeloid and lymphoid cells. AccuCheck beads (Life Technologies) were used to determine absolute numbers of cells by tissue mass normalization. Flow cytometry antibodies are listed in Table 1. 7‐Aminoactinomycin was used to exclude dead cells. Flow cytometry data were collected on a Becton‐Dickenson LSRII analyzer and data were analyzed using FlowJo (TrecStar Software).

### 2.8 Quantitative reverse transcription‐polymerase chain reaction (qRT‐PCR)

RNA was isolated from kidney quarters using Zymo Direct‐zol RNA Miniprep Plus, according to the manufacturer's instructions (Zymo Research). cDNA was made using 1 μg RNA, using the iScript cDNA Synthesis kit instructions (Bio‐Rad Laboratories, Inc.). Five microlitres quantitative polymerase chain reaction (qPCR) reactions were run using BioRad in duplicate using the Applied Biosystems 7900HT Fast Real‐Time PCR Thermal Cycler (Applied Biosystems). Fold changes compared to wild‐type mice were calculated using the comparative Ct (ΔCT) method with *Ubc* as an endogenous control. Primer sequences are listed in Table 2.

**TABLE 2:** qPCR primers sequences utilized in this study

Primers	Forward	Reverse
*Ubc*	5′‐GCCCAGTGTTACCACCAAGAAG‐3′	5′‐GCTCTTTTTAGATACTGTGGTGAGGAA‐3′
*TNF‐α*	5′‐GAGAAAGTCAACCTCCTCTCTG‐3′	5′‐GAAGACTCCTCCCAGGTATATG‐3′
*NGAL*	5′‐CTCAGAACTTGATCCCTGCC‐3′	5′‐TCCTTGAGGCCCAGAGACTT‐3′
*MCP1*	5′‐ACTCACCTGCTGCTACTCAT‐3′	5′‐CTACAGCTTCTTTGGGCAA‐3′
*IL−6*	5′‐ACTCACCTCTTCAGAACGAATTG‐3′	5′‐CCATCTTTGGAAGGTTCAGGTTG‐3′
*IL−1β*	5′‐CAACCAACAAGTGATATTCTCCATG‐3′	5′‐GATCCACACTCTCCAGCTGCA‐3′
*CSF1*	5′‐CGGGCATCATCCTAGTCTTGCTGACTGT‐3′	5′‐ATAGTGGCAGTATGTGGGGGGCATCCTC‐3′
*Fn*	5′‐GCGACTCTGACTGGCCTTAC‐3′	5′‐CCGTGTAAGGGTCAAAGCAT‐3′
*Col1a1*	5′‐GCCAAGAAGACATCCCTGAA‐3′	5′‐GTTTCCACGTCTCACCATTG‐3′
*Col4a1*	5′‐AAGGTGACAAGGGAGAGCAAG‐3′	5′‐CTGTTGGGGCAAAGTCTCCT‐3′
*aKlotho*	5′‐ACAAAGAAGTGGCCGAGAGA‐3′	5′‐CGGTGAAATAGGGCAAAAGA‐3′
*αSMA*	5′‐GACGCTGAAGTATCCGATAGAACAC‐3′	5′‐CCACCATCTCCAGAGTCCAGCACAAT‐3′

Abbreviation: qPCR, quantitative polymerase chain reaction.

### 2.9 Lymphatic density quantification by immunofluorescence

Formalin‐fixed, paraffin‐embedded kidney tissue sections were deparaffinized, rehydrated, and permeabilized with 0.1% Triton solution (BioRad). Sections were blocked with 10% Aqua Block (East Coast Bio) solution in PBS and incubated with antibodies against LEC markers Lymphatic Vessel Endothelial Hyaluronan Receptor 1 (LYVE‐1) and Podoplanin (Table 1). Following fluorescent secondary detection, slides were mounted with ProLong Gold antifade reagent containing 4′,6‐diamidino‐2‐phenylindole (Invitrogen) and imaged using an Olympus BX51 fluorescence microscope with an Olympus Q5 camera. Representative images were captured at 10x magnification using Olympus CellSens imaging software (Olympus). All LYVE‐1+, lumen‐containing lymphatic vessels found around interlobular arteries at the cortex and corticomedullary junction, excluding hilar vessels, by two independent, blinded investigators. For quantification of lymphatics based on podoplanin expression at different time points, five images from predetermined areas within the renal cortex of each kidney section were captured at 10× magnification, with efforts made to exclude tissue defects and glomeruli from the images. The area values measuring the total number of podoplanin + pixels on the images (with the glomerular area removed) were determined using ImageJ (NIH) after setting the threshold for positive endothelium.

### 2.10 Podocyte number quantitation

To identify podocytes, deparaffinized sections were heated in Tris‐EDTA, pH 9.0 for antigen retrieval and labeled with antibodies against podoplanin and Wilms Tumor‐1 (WT‐1) (Table 1). Detection, mounting, and imaging were performed as above. Podocyte counting, cells with a WT1+ nucleus surrounded by podoplanin labeling, was performed as described previously by two blinded investigators (Rutkowski et al., 2013). Because no bias was given to the glomerular area on the section, WT1+ cell numbers per glomerulus ranged from 2 to 15 for normal kidneys. The number counted for each section was averaged across the total number of glomeruli on the section. In disrupted kidneys, when podoplanin expression was reduced in the glomeruli, WT1+ nuclei in correct anatomic positions were deemed as podocytes. WT1+ cell numbers were then divided by the average glomerular cross‐sectional area on the whole kidney section as quantified by blind area measurements performed in ImageJ. The final podocyte counts are therefore reported as WT1+ cells/glomerular area (µm^2^) × 10,000.

### 2.11 Interstitial fibrosis quantification

Picrosirius red staining was performed on renal tissue sections at the CVM Histology Research Laboratory, Texas A&M University. Brightfield cortex images were captured at 10× avoiding tissue edges, medulla, and the renal hilum region. Positive area was quantified using ImageJ software for a range of red hues identified on a positive region of interlobular blood vessels then applied to all images of that staining batch. Total positive pixels are presented as a percent total image area‐averaged first per mouse.

### 2.12 Statistics

All mice received doxycycline to control for any of the target effects and dimerized POD− mice were used as controls in POD‐ATTAC studies. For multiple comparisons across time and genotype a two‐way analysis of variance (ANOVA) (genotype and time) with Tukey post hoc analysis was used. For comparisons of the same genotype over time an ordinary one‐way ANOVA with post‐hoc analysis was used. For qPCR data and day 28 POD‐ATTAC data and picrosirius red analyses, an unpaired *t*‐test with Welch's correction was utilized. The statistical test performed and final number of samples per group are stated in the figure legends. All data are displayed as the mean ± standard deviation. A *p* < 0.05 was considered significant.

## 3 RESULTS

### 3.1 POD‐ATTAC mice demonstrate lymphangiogenesis upon renal injury

**FIGURE 1:**
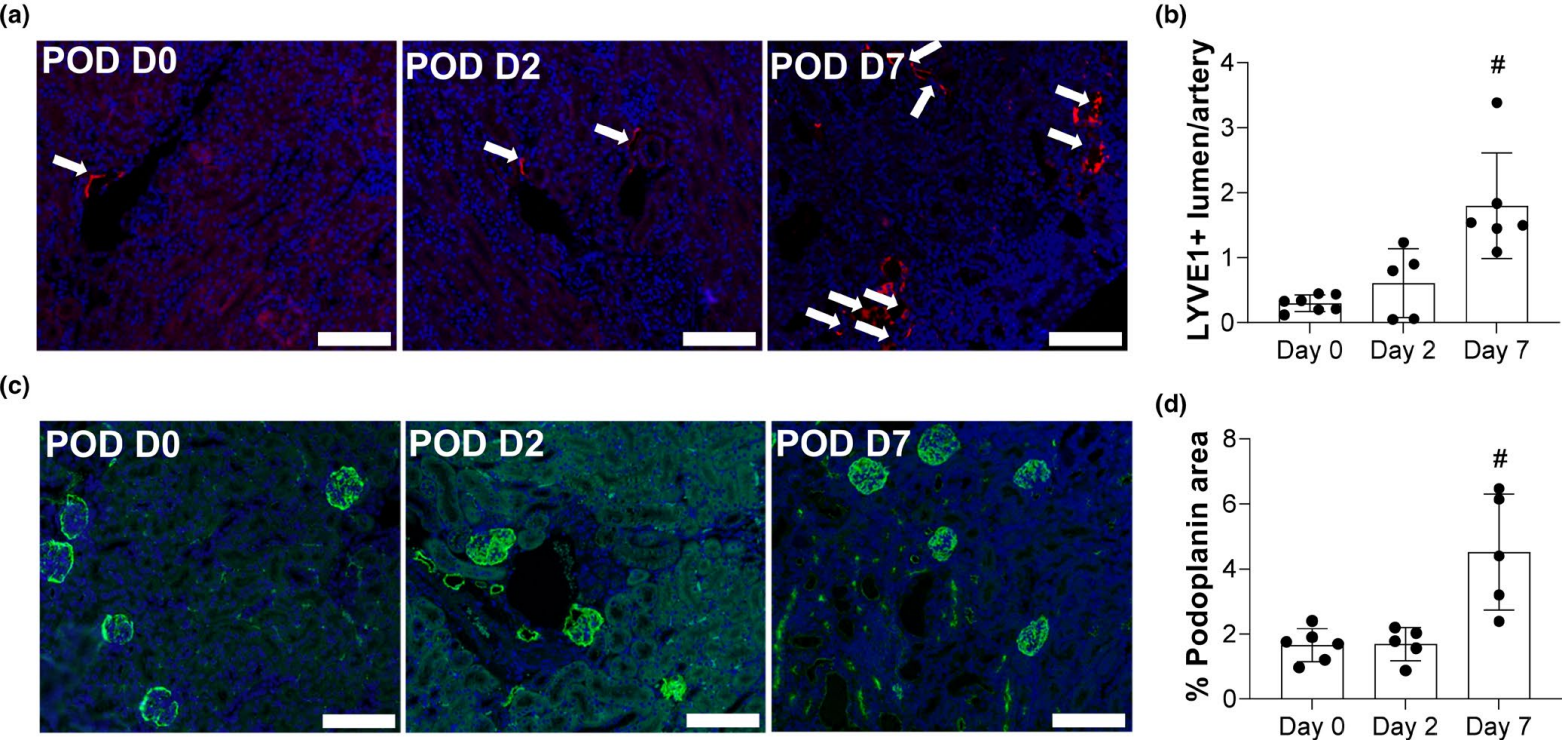
Renal lymphangiogenesis following selective podocyte injury. (a) Immunofluorescence of renal cortical lymphatics labeled for LYVE‐1 (red) and DAPI (blue). Scale bars = 100 µm. (b) Count of LYVE‐1+ lumen/artery (indicated with white arrows) on tissue sections of the cortex. (c) Podoplanin (green) and DAPI (blue) immunofluorescence in the renal cortex. Scale bars = 100 µm. (d) Percent of cortex area positive for podoplanin immunolabeling on tissue sections of the cortex (excluding the glomerular area). Both, LYVE‐1 and podoplanin images were taken at 10× magnification and, for quantification, five different fields/section were imaged. All data are represented as ±SD. Statistical comparisons were made using an ordinary one‐way ANOVA and # indicates *p* < 0.05 over time. ANOVA, analysis of variance; DAPI, 4′,6‐diamidino‐2‐phenylindole; SD, standard deviation

Lymphangiogenesis has been demonstrated in multiple models of renal injury (Creed & Rutkowski, 2021; Zarjou et al., 2019). To determine whether proteinuric injury following podocyte loss induced renal lymphangiogenesis, LYVE‐1, and podoplanin immunolabeling were performed and quantified. In the quiescent kidney, cortical lymphatics are largely localized to the interlobular arteries (Figure 1a) (Russell et al., 2019). Following injury, the number of LYVE1+ vessels counted was significantly higher by day 7 (Figure 1b). Podoplanin labeling was also markedly increased in the cortex; this increased labeling was independent of glomerular podocytes (Figure 1c). Quantification of the total podoplanin+pixels/total cortical area was also measured to be significantly increased by day 7 (Figure 1d). POD‐ATTAC mice, therefore, demonstrate progressive renal lymphangiogenesis within one week of injury induction, similar to the timing demonstrated in AKI models (Zarjou et al., 2019).

### 3.2 Impact of expanded lymphatics on functional response following injury

**FIGURE 2:**
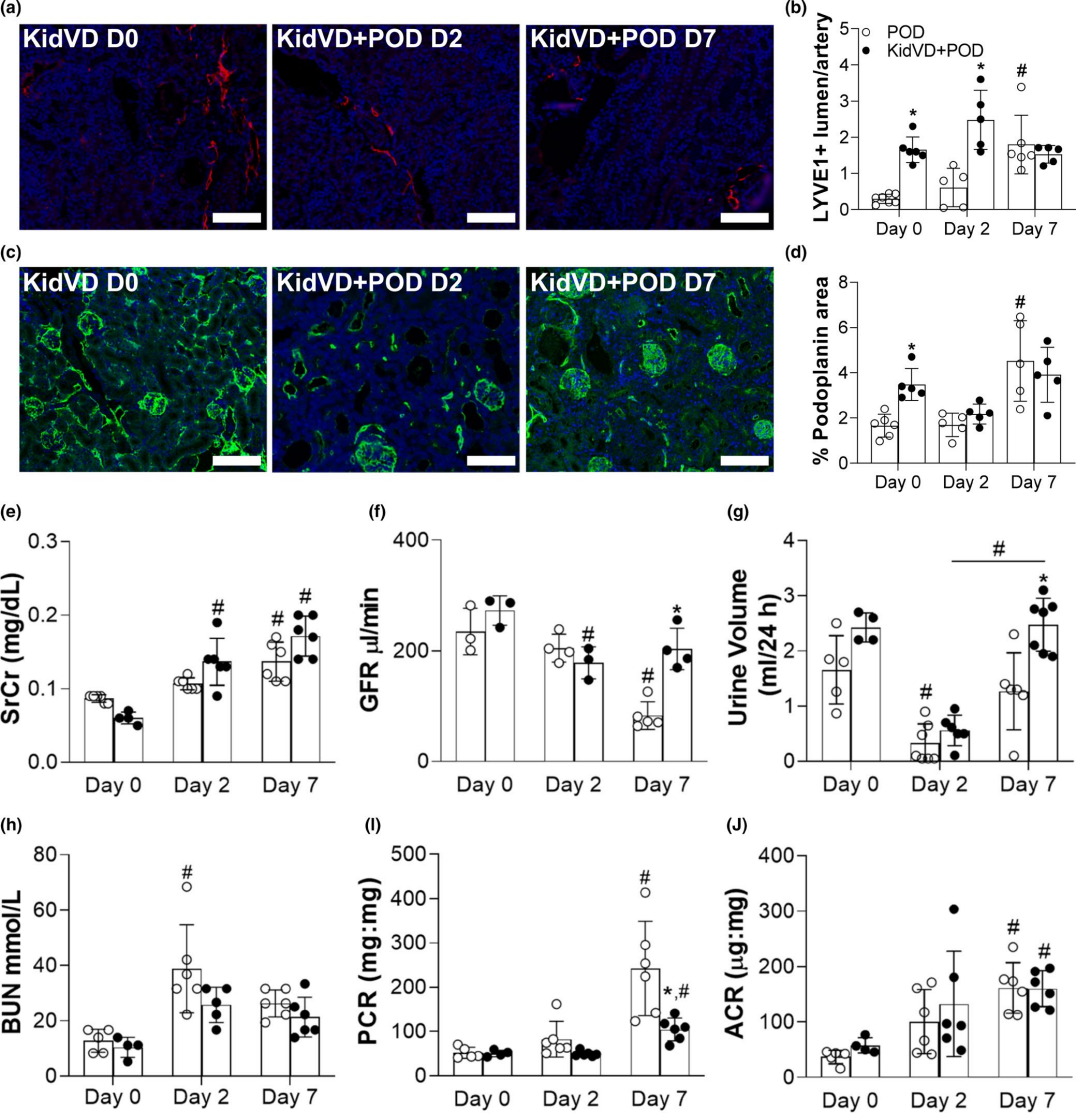
Impact of expanded lymphatics in KidVD mice on the functional response following selective podocyte injury. (a) Immunofluorescence for lymphatics in the renal cortex labeled for LYVE‐1 (red) and DAPI (blue) at 2 and 7 days post‐injury. Scale bars = 100 µm. (b) Count of LYVE‐1+ lumen/artery (indicated with white arrows) on tissue sections of the cortex. (c) Podoplanin (green) and DAPI (blue) immunofluorescence in the renal cortex. Scale bars = 100 µm. (d) Percent of cortex area positive for podoplanin immunolabeling on tissue sections of the cortex (excluding the glomerular area). Both, LYVE‐1 and podoplanin images were taken at 10× magnification and, for quantification five different fields/section were imaged. Functional indicators at 2 and 7 days following dimerizer delivery to KidVD+POD‐ATTAC mice (KidVD+POD) mice and their POD littermates include: (e) serum creatinine (SCr); (f) transcutaneous glomerulation filtration rate (GFR); (g) 24 h urine volume; (h) blood urea nitrogen (BUN); (i) urinary protein:creatinine (PCR); (j) urinary albumin:creatinine (ACR) *n* = 6 POD, 6 KidVD+POD at day 7. Open circles indicate POD and closed circles represent KidVD+POD mice. All data are represented as ±SD. Statistical comparisons were made using two‐way ANOVA with Tukey's correction, *p < 0.05 compares POD to KidVD genotype effect at same time point, #*p* < 0.05 effect from baseline or over time for the same genotype. ANOVA, analysis of variance; DAPI, 4′,6‐diamidino‐2‐phenylindole; SD, standard deviation

To test if lymphatic network expansion could impact the initial response to kidney injury, renal lymphangiogenesis was induced for 3 weeks prior to injury in POD‐ATTAC mice crossed to the KidVD mouse line (KidVD+POD). LYVE1+ lymphatic vessel density within the cortex was significantly greater at the time of injury and 2 days post‐dimerization in KidVD+POD mice (Figure 2a,b). At day 7 KidVD+POD and POD mice demonstrate comparable levels of LYVE1+ vessels due to the significant endogenous lymphangiogenesis that occurs post‐injury in POD mice and VEGF‐D no longer being overexpressed (Figures 1a–d and 2b). We additionally confirmed and quantified increased lymphatic structure density with podoplanin immunolabeling (Figure 2c) and further quantification of the total podoplanin + pixels/total cortical area (Figure 2d).

POD‐ATTAC mice demonstrated clinically relevant indicators of AKI with serum creatinine significantly elevated at days 2 and 7 from baseline values, however, no significant difference in serum creatinine was measured between KidVD+POD mice and their POD littermates (Figure 2e). Transcutaneous GFR measurements, while significantly decreased in POD mice by day 7, were significantly higher in KidVD+POD mice (Figure 2f). At 2 days post‐podocyte injury, 24‐h urine volumes were significantly reduced in both groups but were restored to normal by day 7 in KidVD+POD (Figure 2g). No significant differences in BUN were measured between KidVD+POD mice and their POD littermates (Figure 2h). Renal injury in POD‐ATTAC mice occurs following inducible apoptosis in podocytes leading to rapid and progressive proteinuria that was significant by day 7 though to a significantly lesser extent in KidVD+POD than POD mice (Figure 2i). Increased albuminuria was equivalent across mouse strains, however, in the 7 days following glomerular injury (Figure 2j). The proteinuric time course was thus similar to previous studies using this model (Dizin et al., 2020; Rutkowski et al., 2013). The dimerizer agent utilized to induce the caspase‐8 mediated podocyte apoptosis caused no functional effects in wild‐type mice (Figure S1a–f). POD kidneys do exhibit expression of the AKI marker KIM‐1 by day 7 in both POD and KidVD+POD mice (Figure S2a,b). Overall, KidVD+POD mice demonstrated equivalent functional detriment acutely 2 days after glomerular injury is induced, but the potential for functional preservation compared to POD mice at day 7 in the POD‐ATTAC injury model.

### 3.3 Immune and inflammatory response is similar in KidVD mice following proteinuric injury

**FIGURE 3:**
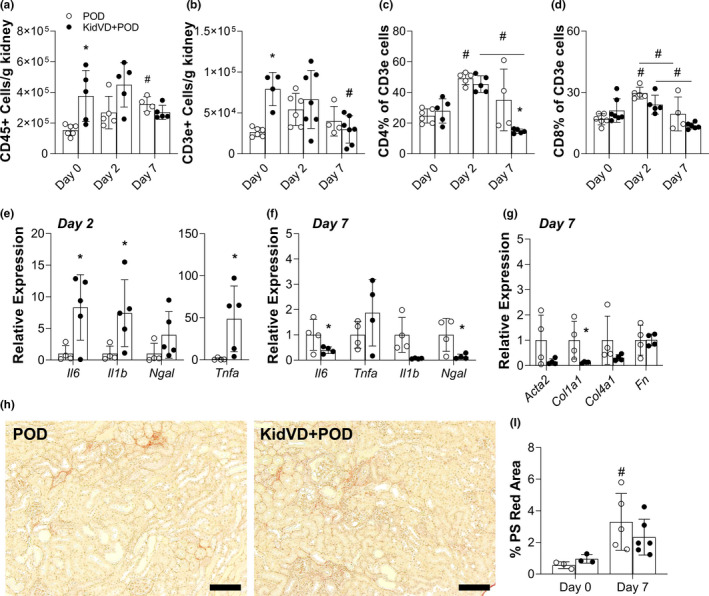
Immune cell and inflammation response in KidVD mice following selective podocyte injury. Flow cytometry analysis of immune cell populations in the kidney at days 0, 2, and 7 following dimerizer delivery to KidVD+POD mice and their POD littermates. (a) Total CD45+ cells/g kidney tissue; (b) total CD3e+ cells; (c) CD4+ T cells % of CD3e+; (d) CD8+ T cells % of CD3e+ (e) mRNA expression levels for the murine genes encoding IL‐6 (*Il6*), IL‐1 beta (*Il1b*), NGAL (*Ngal*), and TNFα (*Tnfa*) in the kidney at day 2 following injury normalized to POD at the same time; note the different y‐axis for *Tnfa*. *n* = 4 POD, 5 KidVD+POD. (f) Cytokine mRNA expression profile in the kidney at day 7 following injury normalized to POD at the same time. *n* = 4 POD, 4 KidVD+POD. (g) mRNA expression for fibrosis of the murine genes encoding α‐SMA (*Acta2)*, Type I collagen (*Col1a1*), Type IV collagen (*Col4a1*), and fibronectin (*Fn*) in the kidney at day 7 following injury normalized to POD at the same time. *n* = 4 POD, 4 KidVD+POD. (h) Picrosirius (PS) red staining on day 7 kidney sections of POD and KidVD+POD mice. Scale bars = 500 µm. (i) Quantified percentage of cortex images positive for intense PS red staining at day 7. *n* = 5 POD, 6 KidVD+POD. Open circles indicate POD and closed circles represent KidVD+POD mice. All data are represented as ±SD. Statistical comparisons were made using two‐way ANOVA with Tukey's correction, *p < 0.05 compares POD to KidVD genotype effect at same time point, #*p* < 0.05 effect from baseline or over time for the same genotype. ANOVA, analysis of variance; DAPI, 4′,6‐diamidino‐2‐phenylindole; SD, standard deviation

The lymphatic system plays an integral role in the inflammatory response following injury (Aspelund et al., 2016). We, therefore, sought to assess if there were immunological differences in KidVD mice that may account for the observed improved functional response. At day 0, KidVD mice had significantly increased total intrarenal immune cells (CD45+) compared to POD controls (Figure 3a). The increased baseline or early inflammation could be a result of VEGF‐D as a chemotactic signaling as previously reported in a model of VEGF‐D overexpression in adipose tissue (Lammoglia et al., 2016). By day 7, immune cell levels were equivalent to POD mice as their inflammation increased (Figure 3a).

We observed significantly increased CD3e+ T cells at day 0, which appeared to account for the bulk of the CD45+ difference in the KidVD model, with more CD3e+ cells at baseline and a significant decline over time in KidVD+POD mice (Figure 3b). The percentage of CD4+ CD3e+ T cells increased from day 0 to 2 for both groups and was significantly reduced by day 7 in KidVD+POD, though this same population remained elevated in POD kidneys (Figure 3c). The percentage of renal CD8+ T cells, however, remained comparable between POD and KidVD+POD mice postinjury (Figure 3d). The numbers of F4/80+Ly6G− macrophages, F4/80−Ly6G+ polymorphonuclear (PMN) cells, B cells (CD19+), and dendritic cells (F4/80−CD11c+) all generally increased following injury in POD‐ATTAC mice, but no significant differences were identified between genotypes (Figure S3a–d, respectively).

Relative RNA expression profiles revealed that the cytokines *Il6*, *ll1b*, and *Tnfa* were all significantly increased in KidVD+POD at day 2 with no significant differences in *Ngal* at this timepoint (Figure 3e). By day 7, however, *Il6* and *Ngal* were found to be significantly reduced compared to POD mice; *Il1b* and *Tnfa*, elevated in KidVD mice at day 2, were unchanged at this timepoint (Figure 3f).

Among markers of fibrosis, at day 7 *Col1a1* was significantly reduced in KidVD+POD mice, though we did not detect any significant differences in *Acta2*, *Col4a1*, or *Fn* (Figure 3g). Picrosirius red staining for collagen deposition did not reveal any visual differences or quantified difference in fibrosis at day 7 between groups (Figure 3h,i). Overall, we identified few significant differences in intrarenal immune cells or inflammation in KidVD+POD mice compared to their controls during early proteinuria. Overall, this data suggest that KidVD+POD mice have an early, and more significant, inflammatory response that may lead to a dampened response and less fibrosis 7 days following injury.

### 3.4 Impact of expanded lymphatics on IRI

**FIGURE 4:**
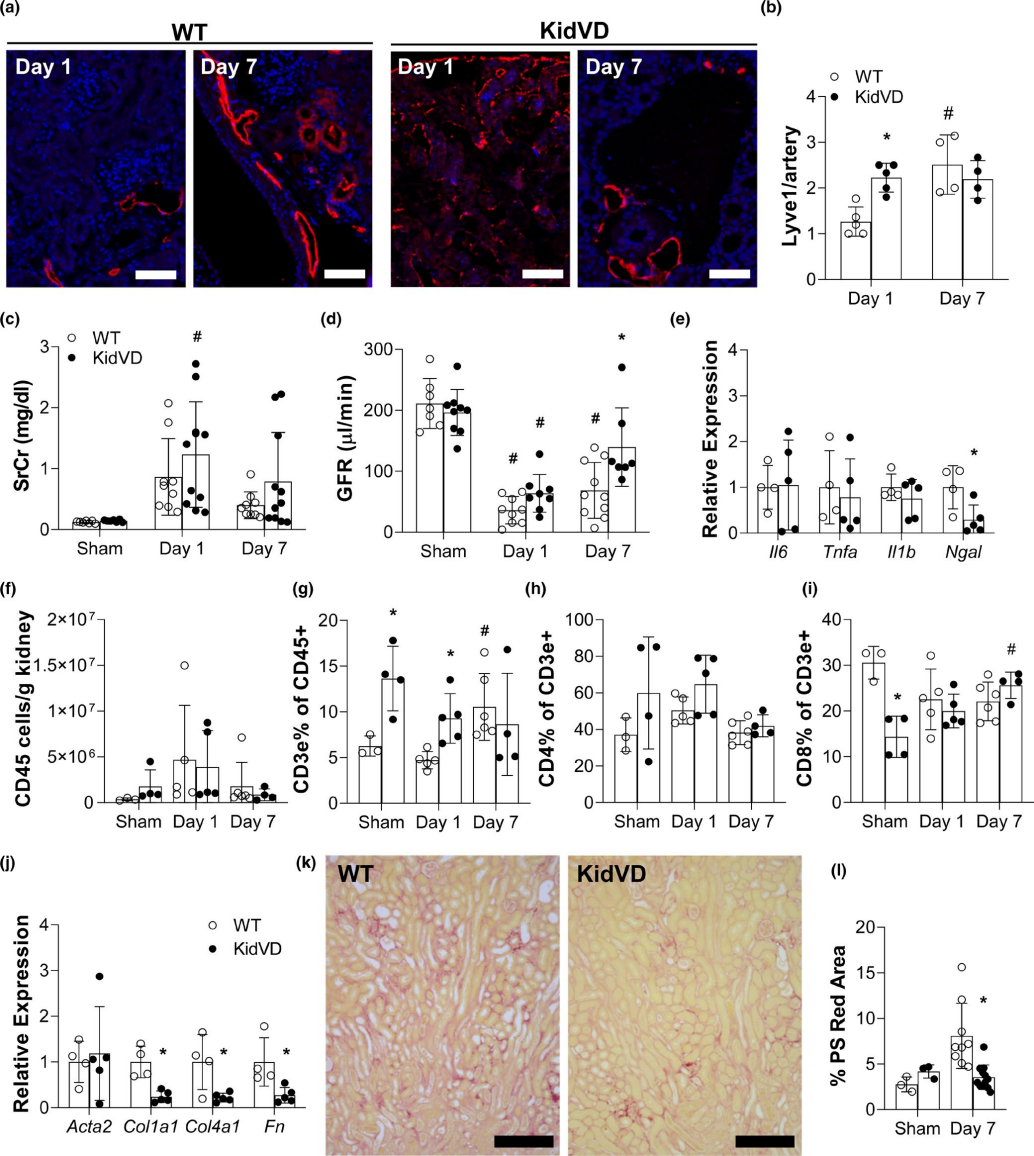
Impact of expanded renal lymphatics on IRI outcomes. (a) Immunofluorescence for lymphatics in the renal cortex labeled for LYVE‐1 (red) and DAPI (blue) 1 and 7 days post‐injury. Scale bars = 100 µm. (b) Count of LYVE‐1+ lumen/artery on tissue sections of the cortex. All the images were taken at 10× magnification and, for quantification, five different fields/section were imaged. (c) Serum creatinine (SCr); and (d) transdermal glomerulation filtration rate (GFR) measured 1 and 7 days following IRI in KidVD mice and their WT littermates. *n* = 7–10 WT and KidVD. (e) mRNA expression levels for the murine genes encoding IL‐6 (*Il6*), IL‐1 beta (*Il1b*), NGAL (*Ngal)*, and TNFα (*Tnfa*). *n* = 4 WT, 5 KidVD. Flow cytometry analysis of immune cell populations in the kidney 1 and 7 days following IRI in KidVD mice and their WT littermates include (f) total CD45+ cells/g kidney tissue; (g) CD3e+ % of CD45+ cells; (h) CD4+ T cells % of CD3e+; (i) CD8+ T cells % of CD3e+; *n* = 3–5 WT, 3–5 KidVD. (j) mRNA expression levels for the murine genes encoding smooth muscle alpha‐actin (*Acta2*), type I collagen (*Col1a1*), type IV collagen (*Col4a1*), and fibronectin (*Fn*) in the kidney at day 7 following injury normalized to POD at the same time. *n* = 4 WT, 5 KidVD. (k) Picrosirius (PS) red staining on day 7 post‐IRI kidney sections of WT and KidVD mice. Bars = 500 µm. (l) Quantified percentage of cortex images positive for intense PS red staining at day 7. *n* = 9 WT, 12 KidVD. Open circles indicate WT and closed circles represent KidVD mice. All data are represented as ±SD. Statistical comparisons were made using two‐way ANOVA with Tukey's correction, *p < 0.05 compares POD to KidVD genotype effect at same time point, #*p* < 0.05 effect over time for the same genotype. ANOVA, analysis of variance; DAPI, 4′,6‐diamidino‐2‐phenylindole; IRI, ischemia‐reperfusion injury; SD, standard deviation; WT, wild‐type

We next tested the effect of lymphatic vessel expansion prior to injury in a bilateral IRI model of AKI. Observed IRI surgical model mortality was comparable between groups (3 of 13 WT and 3 of 17 KidVD across to surgical teams). We first verified increased renal lymphatic density by LYVE1 immunolabeling, which was significantly increased in KidVD+POD mice at day 1 (Figure 4a,b). Similar to the findings in POD‐ATTAC mice, endogenous lymphangiogenesis following IRI reduced this difference by day 7 post‐injury (Figure 4b) (Zarjou et al., 2019). IRI caused marked, though highly variable, rise in serum creatinine in all mice within 1 day of injury that was sustained through day 7 (Figure 4c). GFR was significantly reduced in all mice at day 1 (Figure 4d). At day 7, while wild‐type mice still exhibited significantly reduced GFR, higher GFRs were measured in KidVD mice (Figure 4d).

RNA expression of inflammatory markers IL‐6, TNFa, and Il1‐b were unchanged across genotypes (Figure 4e). Similar to the finding in POD‐ATTAC mice at 7 days postinjury, *Ngal* expression was significantly lower in KidVD mice following IRI (Figure 4e). Flow cytometry analysis identified a trend toward increased CD45+ in sham‐operated KidVD mice and a significant increase of CD3e cells in KidVD mice, when compared to wild‐type sham (Figure 4f,g). We did not detect significant differences in total CD45+ cells between groups after injury (Figure 4f). However, KidVD mouse kidneys contained significantly more CD3e+ T cells in sham‐operated mice and 1 day following injury (Figure 4g). A significant increase in CD3e+ cells in wild‐type mice by day 7 eliminated differences across genotype at this time (Figure 4g). Two‐way ANOVA for time and genotype identified a significantly higher proportion of total T cells to be CD4+ in KidVD mice (*p* = 0.0283), though post‐hoc analysis found no specific differences at each day (Figure 4h). The percent of CD8+ T cells were significantly less in sham‐operated KidVD mice when compared to wild‐type mice, however, this difference was normalized to wild‐type mice by day 7 (Figure 4i). A shift in CD4+ and CD8+ T cell populations was thus similar across two kidney injury models in KidVD mice. Similar to in proteinuric injury, the numbers of F4/80+Ly6G−macrophages, F4/80−Ly6G+ PMN cells, B cells (CD19+), and dendritic cells (F4/80−CD11c+) were increased with inflammation, but comparable between groups over the course of injury (Figure S4a–d, respectively). However, KidVD mice did demonstrate fewer PMN cells at 1 day postinjury than their littermates (Figure S4b).

RNA expression of fibrotic markers *Col1a1*, *Col4a1*, and *Fn* were significantly decreased in KidVD mice compared to their littermates 7 days post‐IRI with *Acta2* unchanged (Figure 4j). In line with the lower relative *Col1a1*, *Col4a1*, and *Fn* RNA expression, picrosirius red staining of collagen in the cortex was significantly reduced in KidVD mice at 7 days post‐IRI (Figure 4k,l). Expanded renal lymphatics prior to injury thus appears to improve the progressive functional and fibrotic response in two different models of kidney injury.

### 3.5 Early lymphangiogenesis improves transitional renal health

**FIGURE 5:**
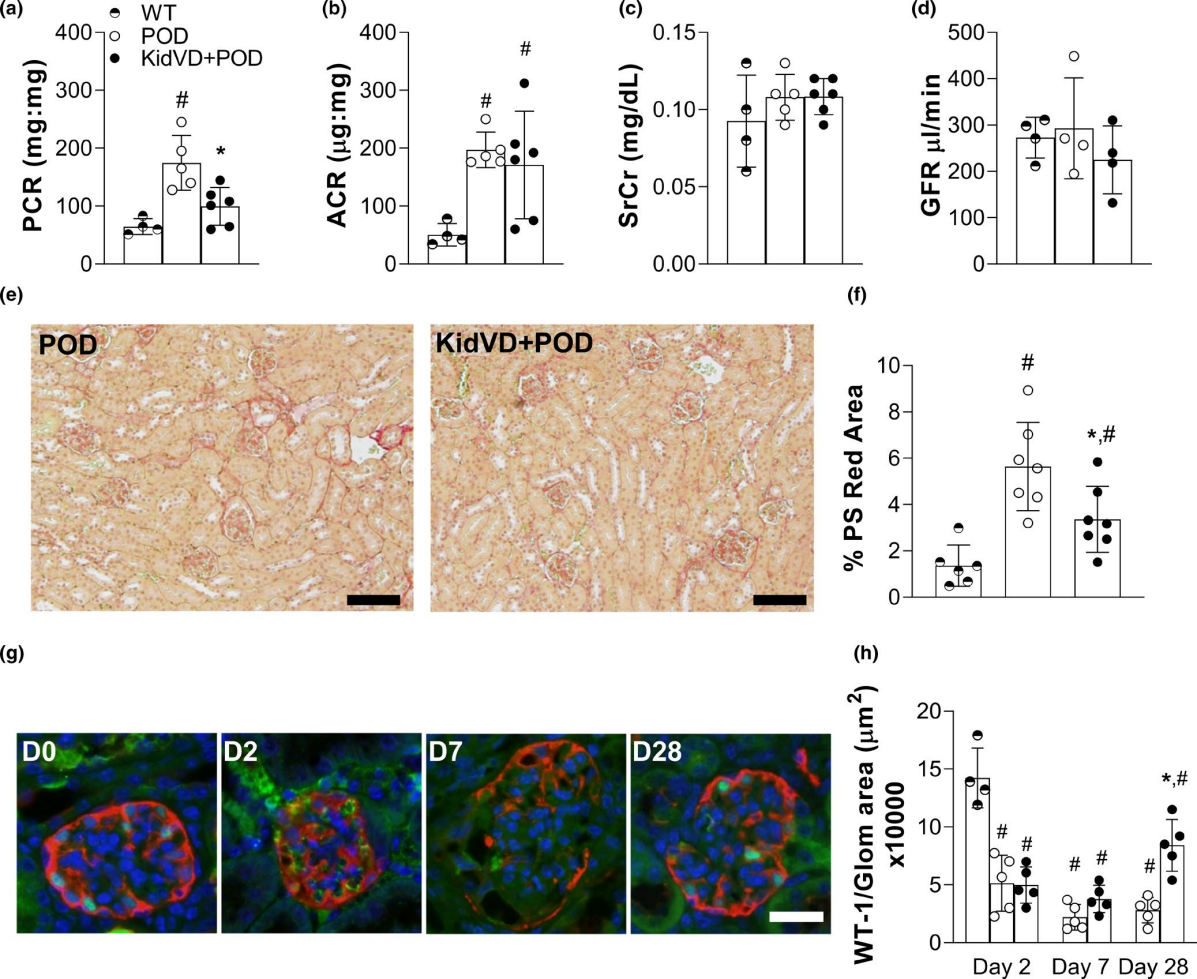
Early lymphangiogenesis improves CKD progression following glomerular injury. Functional indicators 28 days following dimerizer delivery to KidVD+POD mice and their POD and WT littermates include: (a) urinary protein:creatinine (PCR); (b) urinary albumin:creatinine (ACR); (c) serum creatinine (SCr); and (d) transcutaneous glomerulation filtration rate (GFR). *n* = 4–5 POD, 4–5 KidVD+POD. (e) PS red staining on day 28 kidney sections of POD and KidVD+POD mice. Scale bars = 500 µm. (f) Quantified percentage of cortex images positive for intense PS red staining at day 28. *n* = 6 POD, 7 KidVD+POD. (g) Immunofluorescence imaging of WT‐1 for podocyte nuclei (green), podoplanin (red), and DAPI (blue) at 28 days post‐injury. Scale bars = 100 µm. (h) Count of WT1+ podocyte nuclei per glomerulus (WT‐1/glomerular area × 10,000) following injury *n* = 4–5 POD, 5 KidVD+POD. Half open circle represents WT, open circles indicate POD, closed circles represent KidVD+POD mice. WT denotes POD− mouse at 28 days post‐dimerizer delivery. All data are represented as ±SD. Statistical comparisons were made using an unpaired *t*‐test with Welch's correction and *p < 0.05 compares POD to KidVD genotype effect at same time point, #*p* < 0.05 effect over time for the same genotype. ANOVA, analysis of variance; CKD, chronic kidney disease; DAPI, 4′,6‐diamidino‐2‐phenylindole; PS, Picrosirius; SD, standard deviation; WT‐1, Wilms Tumor‐1

Acute kidney injury increases the risk of progression to CKD, which is a major clinical concern. When we followed mice with proteinuric injury for 28 days, we found that proteinuria (Figure 5a) and albuminuria (Figure 5b) were still present in POD‐ATTAC mice, but serum creatinine (Figure 5c) and GFR (Figure 5d) had returned to levels equivalent to uninjured mice. These changes were similar to past work with the model (Rutkowski et al., 2013). In KidVD+POD mice, however, the protein:creatinine ratio was significantly lower than POD mice at 28 days (Figure 5a), though other functional indicators were comparable (Figure 5b–d). Picrosirius red staining area quantitation identified significantly less collagen deposition in KidVD+POD compared to POD mice (Figure 5e,f).

The POD‐ATTAC model has previously demonstrated restoration of podocytes if the injury response was improved (Scarfe et al., 2018). Podocyte numbers were therefore quantified in POD and KidVD+POD mice over time following injury. The loss of WT1+ podocytes was equivalent immediately following injury at days 2 and 7 (Figure 5g,h). In line with the reduced fibrosis, KidVD+mice demonstrated a significant recovery of podocytes by day 28 (Figure 5). Combined, these data suggest that the increased lymphatic density in KidVD+mice prior to injury results in an improved long‐term pathological response.

## 4 DISCUSSION

Lymphatic vessels are key regulators of injury and chronic inflammation. In the current study, we utilized a genetic approach to expand the lymphatic network specifically in the kidney (KidVD) to test the subsequent injury response. Increased renal lymphatic density had little effect on the acute phase response in either injury model, with both demonstrating elevated indicators of inflammation. KidVD mice demonstrated more overall T cells with CD4+ T cell populations increased and fewer CD8+ cells in KidVD mice. KidVD mice demonstrated some improved functional responses and reduced fibrosis by day 7 in the IRI model and day 28 in the POD‐ATTAC injury model. Collectively, our results suggest lymphatics may help to reduce the injury progression to CKD through attenuated renal fibrosis and altered intrarenal immune cell presence.

Lymphatic vessels are key in maintaining tissue homeostasis through exchange and transport of fluids, macromolecules, and immune cells: critical roles during tissue inflammation (Abouelkheir et al., 2017; Aspelund et al., 2016; Huggenberger et al., 2011; Maisel et al., 2017). An expansion of the lymphatic network, both lymphatic vessel hyperplasia and de novo lymphangiogenesis, are often associated with inflammation. Under normal physiological conditions, the cortical lymphatic network is not extensive, with lymphatics following along larger blood vessels rather than existing throughout the peritubular interstitium (Jafree & Long, 2020; Russell et al., 2019). However, following AKI, the lymphangiogenic ligands VEGF‐C and VEGF‐D are significantly elevated within days and lymphatic density is increased over time throughout the kidney, findings recapitulated in both human biopsy samples and several mouse models (Hasegawa et al., 2017; Kasinath et al., 2019; Kinashi et al., 2017; Pei et al., 2019; Sakamoto et al., 2009; Zarjou et al., 2019). Similar to these findings, our study identified a significant increase in lymphatic density in response to a progressive proteinuric kidney injury or IRI by day 7 post‐injury. Lymphatic vessels and renal lymphangiogenesis following kidney injury have been reported to be beneficial in some models by reducing fibrosis and increasing functional recovery, while others have reported detrimental injury outcomes due to lymphatic propagation of a pro‐inflammatory innate immune response (Creed & Rutkowski, 2021).

Lymphatic drainage from the periphery to the lymph nodes is necessary for adaptive immune responses and aids in self‐antigen tolerance (Hirosue et al., 2014; Lund & Swartz, 2010; Maisel et al., 2017; Vokali et al., 2020). From that perspective, the use of neutralizing antibodies against lymphatic chemokines or lymphadenectomy was successful in reducing immune trafficking to the renal lymph node and improved functional outcomes in AKI and crescentic glomerulonephritis (Kasinath et al., 2019; Pei et al., 2019). Increasingly, however, active roles of LECs in peripheral immunomodulation have been identified. Lymphatic vessels and LECs help to maintain tolerogenic cross‐presentation of self‐antigen and help to limit CD8+ T cell accumulation in the periphery (Hirosue et al., 2014; Lane et al., 2018). For example, disruption of LEC signaling to the immune environment increases pathogenic Th17 tissue infiltration and further propagates pro‐inflammatory conditions (Harle et al., 2021). In the present study, we tested how increased renal lymphatic density prior to the onset of AKI would impact renal outcomes and found that, while the acute response was largely unchanged, the hallmarks of CKD progression such as kidney function and fibrosis were improved. In KidVD mice, increased presence of total T cells at baseline and immediately post‐injury suggest that increased renal lymphatic density is actively fulfilling an immunomodulatory role without injury stimulus. This suggests that increased lymphatics may increase immune education prior to injury—through antigen trafficking or direct lymphatic‐immune interactions—priming a more beneficial adaptive immune response. This would be in contrast to a blocking approach that may initially reduce an innate or autoimmune response to injury (Kasinath et al., 2019; Pei et al., 2019). The specific immune cell populations present in KidVD mice, in quiescence and during early injury, may identify whether this lymphatic communication mechanism is part of the response in this model.

Immune infiltration is common following injury and many immune cell types have been identified as part of the pathogenesis of AKI (Singbartl et al., 2019). T cells play an important role in AKI pathology, both toward protecting and promoting the injury response (Kinsey & Okusa, 2014). Multiple mechanisms have reflected how T lymphocytes can mediate renal dysfunction and injury, with evidence indicating that CD4+ T cells often mediate these pathways (Akcay et al., 2011; Ko et al., 2011; Nozaki et al., 2011). In fact, the adoptive transfer of CD4+ T cells reduced the severity of injury, immune cell infiltration, and fibrotic response in a model of folic acid‐induced AKI (Bajwa et al., 2017). Another study further supports a beneficial role for intrarenal CD4+ T cell presence and reported that infusion of CD4+CD25+ Tregs at day 1 post‐IRI had a protective role by modulating pro‐inflammatory cytokines (Gandolfo et al., 2009). Our study utilized the POD‐ATTAC model, which has been previously reported as a model of focal segmental glomerulosclerosis (FSGS), otherwise known as nephrotic syndrome (Dizin et al., 2020; Rutkowski et al., 2013). The use of this model provides a genetic tool to investigate progressive renal disease initiated from a one‐time isolated glomerular injury. Elevated CD3+ T cells have been reported in the kidney biopsies of human FSGS patients compared to controls and high levels of CD8+ T cells with reduced CD4+ T cells found in FSGS patients compared to controls (Kronbichler et al., 2016). Our injury models mirrored these results and demonstrated that increased lymphatics may beneficially alter the CD4:CD8 ratio. While a specific mechanism was not further interrogated in this study, we suggest that LECs play important roles in early immunomodulation and likely contribute to the adaptive immune response.

A partial restoration of podocyte numbers over time is likely to be indicative of the overall improved injury response in KidVD mice. POD‐ATTAC mice have previously demonstrated recovery of podocyte numbers following injury in a mouse model of adiponectin over‐expression (Rutkowski et al., 2013). Less fibrosis was observed in the mice in these studies; however, it is unclear whether podocyte recovery plays an important role in the observed renoprotective outcomes. Podocyte survival has previously been linked to VEGF‐C signaling and, furthermore, a recent study demonstrated that podocyte‐specific overexpression of VEGF‐C resulted in an improvement in albuminuria in a diabetic mouse model (Muller‐Deile et al., 2009; Onions et al., 2019). In the current study, however, the inducible transgenic overexpression of VEGF‐D was stopped prior to the induction of injury and the same number of podocytes were lost at days 2 and 7, making direct VEGF‐D‐podocyte effects unlikely. A direct mechanism cannot, however, be fully ruled out. Human and murine VEGF‐C has the ability to signal through VEGFR‐2, which is expressed by many renal cells. However, murine VEGF‐D is unable to bind VEGFR‐2 and thus only signals through VEGFR‐3 (Baldwin et al., 2001). Though the induction of VEGF‐D was stopped before injury in both POD‐ATTAC and IRI mice, VEGF‐D effects could also potentially result from signaling on other cells expressing VEGFR‐3 in the kidney (Kenig‐Kozlovsky et al., 2018). The recruitment of immune cells, immune cell‐LEC interactions, subsequent lymphangiogenesis, VEGFR‐3‐signaling, and their cumulative effects on the AKI are therefore likely time‐ and model‐dependent as to their harm or utility.

The current study demonstrates that KidVD mice demonstrate equivalent renal injuries to controls during the acute phase but have reduced fibrosis and improved functional recovery overtime. Fibrosis is indicative of CKD progression and genetic models targeting the process have demonstrated improved function following AKI (Chung et al., 2018; Huffstater et al., 2020). We recognize, however, that a direct mechanism was not determined here, but will be the focus of future studies. Previous work with POD‐ATTAC mice was on an FVB background with different dimerizer dosing, so some functional readouts, such as GFR, were altered more strongly than before. While transdermal GFR measurements allow for real time measurement of renal filtration we recognize that the current literature does not have studies validating the accuracy of this method in states of edema or ascites. Additional functional readouts such as urine volumes and serum creatinine generally support conclusions drawn from the transdermal GFR data. Overall, the totality of our data from two models demonstrates that increased lymphatic vessel density is beneficial in renal recovery post‐injury.

Lymphatic vessels and lymphangiogenesis are reported to play multiple beneficial and detrimental roles following AKI, therefore the precise role of lymphatics in kidney injury remain unclear (Creed & Rutkowski, 2021). The current study demonstrates a significant positive impact of increased lymphatic density in the kidney prior to injury that improves functional recovery post‐AKI. Future studies examining the impact of augmenting renal lymphatic density at different points throughout injury and its impact on immune cell phenotypes will further define the roles of lymphatics in AKI and the AKI‐to‐CKD progression.

## CONFLICT OF INTEREST

The authors have no conflicts to disclose.

## AUTHOR CONTRIBUTIONS

Gaurav Baranwal, Heidi A. Creed, and Laurence M. Black designed the experiments, carried them out, analyzed data, and contributed to the writing of the manuscript. Alexa Auger, Alexander M. Quach, Rahul Vegiraju, and Han E. Eckenrode, helped to design experiments, carry them out, and analyze data. Anupam Agarwal and Joseph M. Rutkowski conceived the experiments, aided in their design, and analyzed data. Joseph M. Rutkowski prepared the manuscript with assistance from all authors. All authors read and approved the manuscript.

## Supporting information



Fig S1‐S4
