# Supplementary material for: Expanded renal lymphatics improve recovery following kidney injury
Source: Physiol Rep. 2021 Nov 21;9(22):e15094. doi: 10.14814/phy2.15094 (PMC8606868; doi:10.14814/phy2.15094)
Supplement: Supplementary file 1 — Fig S1‐S4 [file PHY2-9-e15094-s001.pdf]

Supplementary Figures for

Expanded renal lymphatics improve recovery following kidney injury

Gaurav Baranwal[1#], Heidi A. Creed[1#], Laurence M. Black[2,3#], Alexa Auger, Alexander M. Quach, Rahul Vegiraju, Hannah E. Eckenrode[2,3], Anupam Agarwal[2,3,4], Joseph M. Rutkowski[1]*

1: Division of Lymphatic Biology, Department of Medical Physiology, Texas A&M University College of Medicine, Bryan, TX 77807 USA
2: Department of Medicine, University of Alabama at Birmingham, Birmingham, AL, 35294, USA.
3: Nephrology Research and Training Center, University of Alabama at Birmingham, Birmingham, AL, 35294, USA.
4: Department of Veterans Affairs, Birmingham, AL, USA
#: denotes equal contribution

*Correspondence:
Joseph M Rutkowski
Texas A&M University College of Medicine
8447 Riverside Parkway
Bryan, TX 77807
United States
Ph: +1-979-436-0576
Fax:+1-979-436-9294
Email: rutkowski@tamu.edu

Short title: Renal lymphatics in kidney injury

Supplementary Figure 1

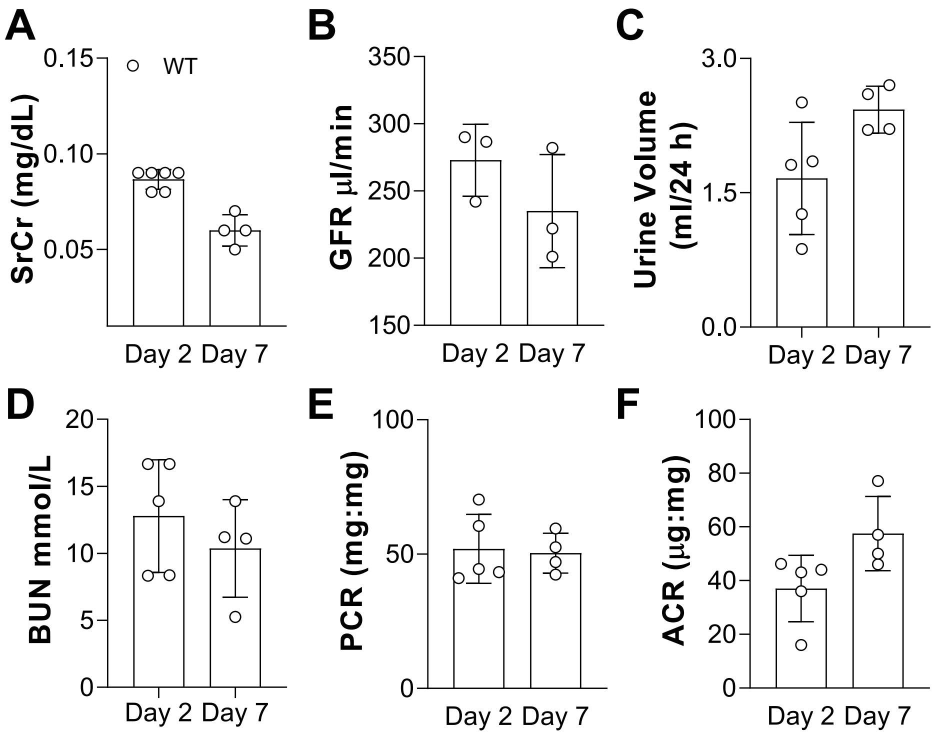

Supplementary Figure 1 Dimerizer effect on WT mice. Functional indicators at 2 and 7 days following dimerizer delivery to wild type littermates include: (A) serum creatinine (SCr); (B) transcutaneous glomerulation filtration rate (GFR); (C) 24 hour urine volume; (D) blood urea nitrogen (BUN); (E) urinary protein:creatinine (PCR); (F) urinary albumin:creatinine (ACR) n=5 (day 2), 4 (day 7). n=5 (day 2), n=4 (day 7) for all except GFR n=3 at both time points. Statistical comparisons were made using an unpaired t-test with Welch's correction.

Supplementary Figure 2

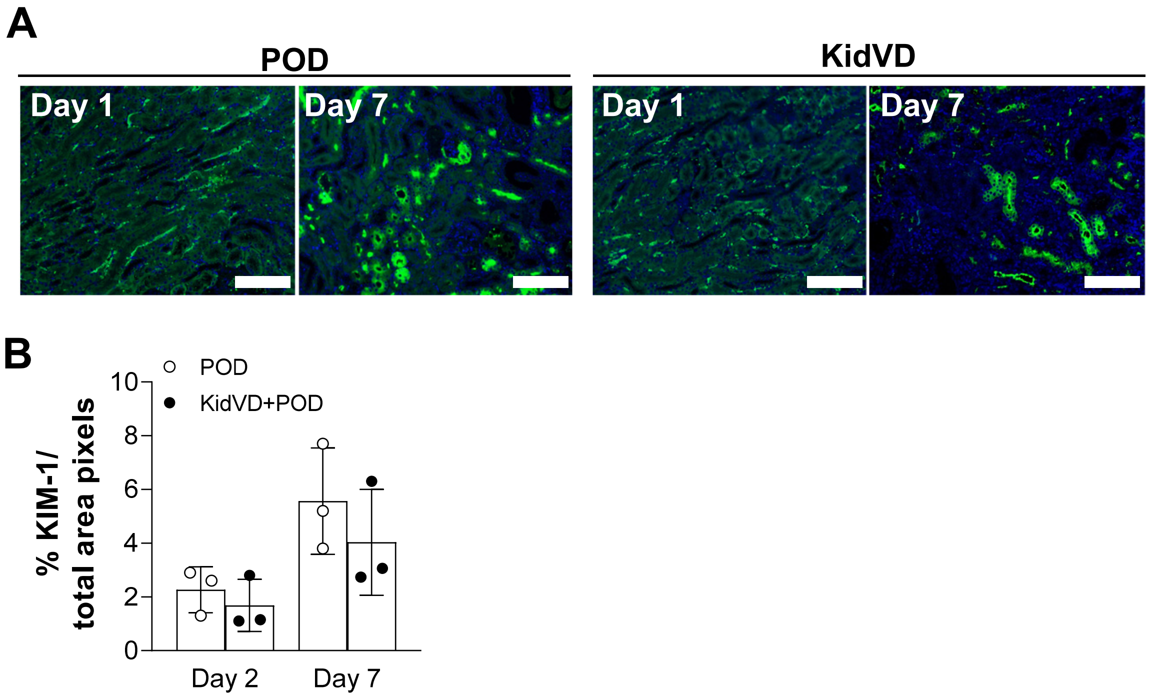

Supplementary Figure 2 Kim1 staining indicates a similar extent of injury in POD vs. KidVD+POD. (A) Immunofluorescence imaging of Kidney injury molecule-1 (KIM-1), for Kim1 (green), and DAPI (blue) at 2 and 7 days post-injury. Scale bars = 100 μm. (B) Percent of area positive for KIM-1 immunolabeling on tissue. The images were taken at 10X magnification and, for quantification, 5 different fields/section were imaged. (n=3) Statistical comparisons were made using two-way ANOVA with Tukey's correction, *p<0.05 compares POD to KidVD genotype effect at same time point, #p<0.05 effect over time for the same genotype.

Supplementary Figure 3

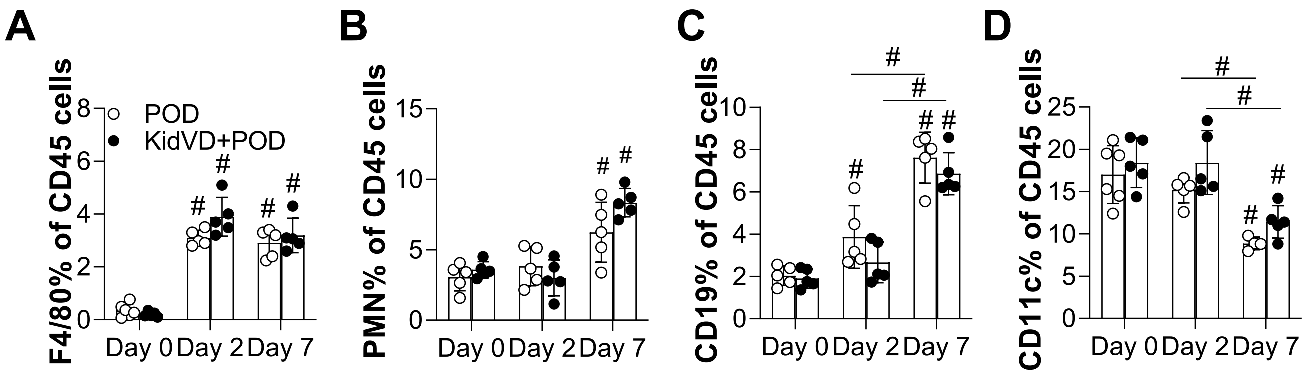

Supplementary Figure 3 Immune cell populations in POD-ATTAC mice. (A) F4/80+Ly6G- macrophages % of CD45+ cells; (B) F4/80-Ly6G+ polymorphonuclear (PMN) cells % of CD45+ cells; (C) CD19+ % of CD45+ cells; and (D) CD11c+ % of CD45+ cells. n=4 POD, 5 KidVD+POD. Statistical comparisons were made using two-way ANOVA with Tukey's correction, *p<0.05 compares POD to KidVD+POD genotype effect at same time point, #p<0.05 effect from baseline or over time for the same genotype.

Supplementary Figure 4

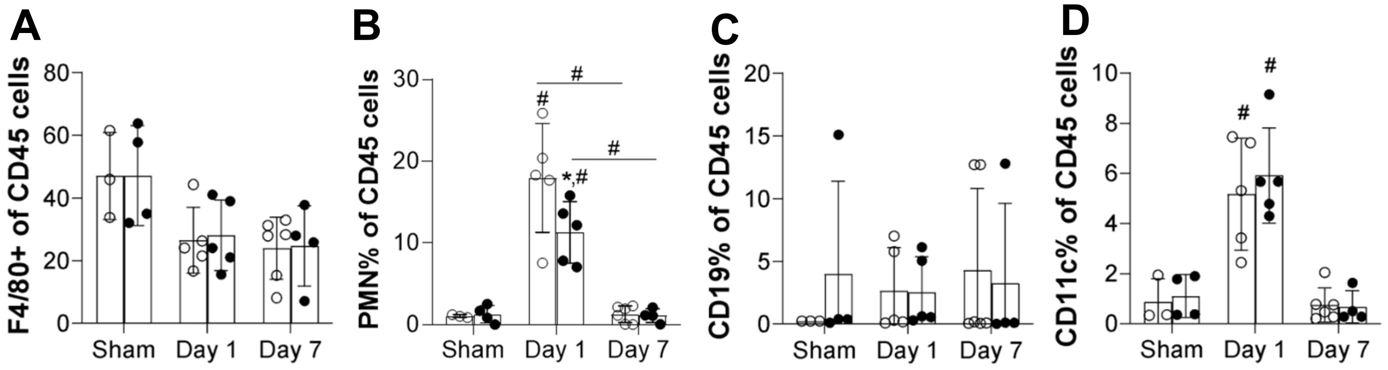

Supplementary Figure 4 Immune cell populations in IRI mice. (A) F4/80+Ly6G- macrophages % of CD45+ cells; (B) F4/80-Ly6G+ polymorphonuclear (PMN) cells % of CD45+ cells; (C) CD19+ % of CD45+ cells; and (D) CD11c+ % of CD45+ cells. Statistical comparisons were made using two-way ANOVA with Tukey's correction, *$p<0.05$ compares WT to KidVD genotype effect at same time point, #$p<0.05$ effect from baseline or over time for the same genotype.
